# Uric Acid as a Risk Factor for Cardiovascular Disease and Mortality in Overweight/Obese Individuals

**DOI:** 10.1371/journal.pone.0059121

**Published:** 2013-03-22

**Authors:** Helle Skak-Nielsen, Christian Torp-Pedersen, Nick Finer, Ian D. Caterson, Luc Van Gaal, W. Philip T James, Aldo Pietro Maggioni, Arya M. Sharma, Walmir Coutinho, Charlotte Andersson

**Affiliations:** 1 Department of Cardiology, Gentofte University Hospital, Hellerup, Denmark; 2 UCL Institute of Cardiovascular Science, London, United Kingdom; 3 Boden Institute, University of Sydney, Sydney, New South Wales, Australia; 4 Department of Endocrinology, Diabetology and Metabolic Diseases, Antwerp University Hospital, Antwerp, Belgium; 5 London School of Hygiene and Tropical Medicine, London, United Kingdom; 6 Associazione Nazionale Medici Cardiologi Ospedalieri (ANMCO) Research Center, Florence, Italy; 7 University of Alberta, Edmonton, Canada; 8 Catholic University of Rio de Janeiro, Rio de Janeiro, Brazil; Virginia Commonwealth University, United States of America

## Abstract

**Background:**

The predictive value of serum uric acid (SUA) for adverse cardiovascular events among obese and overweight patients is not known, but potentially important because of the relation between hyperuricaemia and obesity.

**Methods:**

The relationship between SUA and risk of cardiovascular adverse outcomes (nonfatal myocardial infarction, nonfatal stroke, resuscitated cardiac arrest or cardiovascular death) and all-cause mortality, respectively, was evaluated in a post-hoc analysis of the Sibutramine Cardiovascular OUTcomes (SCOUT) trial. Participants enrolled in SCOUT were obese or overweight with pre-existing diabetes and/or cardiovascular disease (CVD). Cox models were used to assess the role of SUA as an independent risk factor.

**Results:**

9742 subjects were included in the study; 83.6% had diabetes, and 75.1% had CVD. During an average follow-up time of 4.2 years, 1043 subjects had a primary outcome (myocardial infarction, resuscitated cardiac arrest, stroke, or cardiovascular death), and 816 died. In a univariate Cox model, the highest SUA quartile was associated with an increased risk of cardiovascular adverse outcomes compared with the lowest SUA quartile in women (hazard ratio [HR]: 1.59; 95% confidence interval [CI]: 1.20–2.10). In multivariate analyses, adjusting for known cardiovascular risk factors the increased risk for the highest SUA quartile was no longer statistically significant among women (HR: 0.99; 95% CI: 0.72–1.36) nor was it among men. Analyses of all-cause mortality found an interaction between sex and SUA. In a multivariate Cox model including women only, the highest SUA quartile was associated with an increased risk in all-cause mortality compared to the lowest SUA quartile (HR: 1.51; 95% CI: 1.08–2.12). No relationship was observed in men (HR: 1.06; 95% CI: 0.82–1.36).

**Conclusion:**

SUA was not an independent predictor of cardiovascular disease and death in these high-risk overweight/obese people. However, our results suggested that SUA was an independent predictor of all-cause mortality in women.

## Introduction

The association of gout with cardiovascular disease, first described in the 19^th^ century was thought to be an association, rather than a cause, of cardiovascular disease. More recent evidence from cohort studies suggests a causal relationship between hyperuricaemia and risk of adverse cardiovascular events [1]–[4]. Some studies have only been able to show this for women [5], [6], while others have failed to demonstrate this association after controlling for various well known atherosclerotic risk factors [7], [8].

Because hyperuricemia is closely related to obesity, hypertension [9], and dyslipidaemia [10], it has been difficult to establish whether or not an independent association between uric acid and cardiovascular disease exists. Elevated uric acid might merely represent an indirect marker of the metabolic syndrome. To examine this question several studies have tested the effect of allopurinol treatment on the risk of adverse cardiovascular events, and high dose allopurinol seems to be associated with better survival than low dose allopurinol, both in patients with congestive heart failure [11] and in a general hospital patient population [12].

Most studies investigating the relationship between uric acid and cardiovascular disease have included either healthy populations or patients with hypertension or congestive heart failure. Hyperuricaemia does not form part of current definitions of the cardio-metabolic syndrome [13]–[15], a syndrome closely associating overweight and obesity with adverse cardiovascular outcomes. We have investigated the relationship between initial serum uric acid concentration and risk of developing new cardiovascular events or mortality in a population of nearly 10,000 overweight or obese, cardiovascular high risk patients.

## Methods

The present analysis used data from the prospective SCOUT (Sibutramine Cardiovascular OUTcomes) trial, a randomized, double-blinded, placebo-controlled, multicentre clinical study with sibutramine, a sympathomimetic agent used for weight loss. SCOUT was conducted from January 2003 through March 2009 in 16 different countries in Europe, Australia and Latin America. The primary study endpoint comprised a composite of nonfatal myocardial infarction, nonfatal stroke, resuscitation after cardiac arrest, and cardiovascular death (primary outcome events). A secondary endpoint was all-cause mortality.

The details regarding the SCOUT trial have been described elsewhere [16]. In brief, all patients in the trial were 55 years of age or older, had body-mass index (BMI)>27 kg/m^2^ and <45 kg/m^2^, or a BMI>25 kg/m^2^ and <27 kg/m^2^ plus a waist circumference of at least 102 cm/88 cm (men/women). Another criterion was a history of cardiovascular disease (defined as a history of coronary artery disease, peripheral arterial occlusive disease or stroke), or diabetes mellitus type 2 with at least one other cardiovascular risk factor (defined as hypertension, dyslipidaemia, current smoker, or diabetic nephropathy), or both. Because of a lower than expected rate of primary outcome events, the inclusion criteria changed after 15 months so that only the highest risk patients were enrolled, i.e. patients with both a history of cardiovascular disease *and* diabetes mellitus type 2 with one other cardiovascular risk factor.

Medical conditions such as diabetes, hypertension and dyslipidaemia were to be treated as indicated by national guidelines available at the time of this trial. Blood tests, including uric acid, were performed at randomization baseline and annually thereafter. All blood tests were performed fasting, and were analysed in a certified central laboratory.

Subjects were followed until their final visit, which was between November 2008 and March 2009. For those subjects who prematurely discontinued the study drug, follow up data were obtained until March 2009 and analysed by the intention-to-treat principle. For each of the separate the endpoints, patients were censored after their first event.

We have divided the subjects into quartiles of uric acid concentrations at baseline with separate cut off-points for men and women, because women have lower SUA levels than men on average. We investigated the overall rate of primary outcome events, the rate of each component of the composite primary outcome, and the rate of all-cause mortality.

### Ethics

This study (Clinical Trial registration Number NCT00234832) was approved be relevant local ethics committees and conducted according to the Declaration of Helsinki. All participating subjects had given their written informed consent prior to enrolment.

### Statistics

Differences in continuous and discrete variables were compared using analysis of variance and the Cochran-Armitage trend test, respectively. Survival distribution function curves were constructed using Kaplan-Meier methods.

We used Cox proportional hazard regression models to perform univariate and multivariate analyses, stratifying for sex and adjusting for the following factors: age, cholesterol, high-density lipoprotein cholesterol (HDL-C), low-density lipoprotein cholesterol (LDL-C), BMI, glucose, triglycerides, creatinine, history of only diabetes mellitus/diabetes mellitus and cardiovascular disease/only cardiovascular disease, congestive heart failure, systolic blood pressure, diastolic blood pressure, waist-hip ratio, tobacco use, alcohol use, use of diuretics, use of beta-blockers, use of statins, use of aspirin and randomized treatment (placebo vs. sibutramine). Hazard ratios (HR) and their corresponding 95% confidence intervals (CI) for uric acid quartiles were constructed within the framework of the Cox models. Two-way interactions between baseline uric acid level (i.e. values at the time of randomization) and age, sex, BMI, glucose level, congestive heart failure, diabetes mellitus, systolic and diastolic blood pressure, waist-hip ratio, use of diuretics, and randomized treatment were tested in the Cox regression models. The effect of changes in uric acid level during the trial was also assessed. To ensure that changes in uric acid levels during follow-up did not influence the initial findings a time-dependent multivariable Cox regression model was performed. In this model, uric acid levels (as a class variable) and systolic blood pressure levels (as a continuous variable) were updated at an annual basis after study baseline. Due to concerns for close correlation, baseline values of these variables were not included in the model.

We assessed the proportional hazards assumption both visually using log-log curves and quantitively testing Schoenfelds residuals for time-dependency. Model assumptions including linearity, proportional hazards and interactions were tested and found valid unless otherwise described. All calculations were done in SAS, version 9.2 (SAS institute, Cary, North Carolina). The level for statistical significance was set at a p-value<0.05.

## Results

Of the 9,804 subjects who were randomised, 9,742 had serum uric acid concentration measured at baseline and formed the population used in the present analyses. The total follow-up involved 41,195 person years, with an average follow-up time of 4.2 years per subject. There were 816 deaths; 449 (55%) were due to cardiovascular causes. Overall, 1043 primary outcome events were reported (355 nonfatal myocardial infarctions, 221 nonfatal strokes, and 18 resuscitated cardiac arrests). At baseline, 57.6% were men, 83.6% had diabetes, and 75.1% had CVD; mean±standard deviation age, BMI, and SUA were 63.2±6.1 years, 34.5±4.5 kg/m^2^, and 364.7 µmol/l (range 89.0–867.0 µmol/l), respectively. Demographic and baseline characteristics for subgroups defined by gender and quartiles of baseline uric acid concentrations are shown in Table 1 and 2, respectively.

**Table 1:** Baseline characteristics of the study population.

	Overall	Men	Women	P-value
Number of patients	9742	5612	4130	<0.0001
Age (years)	63.2 (6.1)	63.3 (6.1)	63.2 (6.1)	0.58
History of CVD (%)	75.0	83.1	64.0	<0.0001
Diabetes (%)	83.6	81.5	86.4	<0.0001
Total Cholesterol (mmol/l)	194.7 (44.2)	185.8 (41.9)	206.9 (44.3)	<0.0001
Triglycerides (mmol/l)	200.1 (123.0)	205.4 (134.6)	193.0 (104.7)	0.026
Blood glucose(mmol/l)	8.5 (3.2)	8.4 (3.0)	8.6 (3.3)	0.047
HgbA1c*	7.5 (1.4)	7.5 (1.4)	7.6 (1.5)	0.004
BMI (kg/m2)	34.5 (4.5)	33.6 (4.1)	35.6 (4.8)	<0.0001
Systolic bloodpressure (mmHg)	138.2 (12.7)	137.4 (13.0)	139.3 (12.4)	<0.0001
Diast. blood pressure (mmHg)	77.8 (8.4)	78.3 (8.4)	77.2 (8.4)	<0.0001
Diuretics users (%)	47.6	44.3	52.1	<0.0001
Beta-blocker users (%)	61.4	65.1	56.3	<0.0001
Statin users (%)	66.5	70.5	61.1	<0.0001
Aspirin users (%)	78.6	85.9	68.6	<0.0001
Sibutramine users (%)	50.0	49.6	50.5	0.39
Smokers (%)	58.2	74.6	35.9	<0.0001
Alcohol users (%)	61.6	77.6	39.9	<0.0001
Primary outcomeevent (%)	10.7	12.5	8.2	<0.0001
All-cause mortality (%)	8.4	9.8	6.5	<0.0001

Continuous variables are presented as means (SD). Abbreviations: BMI = body mass index. Diast. = diastolic. P-values were obtained by analysis of variance for continuous variables and by Cochran-Armitage trend test for discrete variables.

* HbA1c was only measured in diabetics.

**Table 2:** Baseline characteristics in men and women stratified by SUA quartiles.

	Men					Women				
	Q1	Q2	Q3	Q4	P-value	Q1	Q2	Q3	Q4	P-value
Number of patients	1406	1423	1385	1398		1056	1028	1017	1029	
Age (years)	62.8 (6.0)	63.3 (6.2)	63.6 (6.2)	63.5 (6.2)	0.004	62.6 (5.9)	63.1 (5.9)	63.1 (6.2)	63.9 (6.2)	<0.0001
History of CVD (%)	77.9	81.5	85.6	87.4	<0.0001	57.8	63.7	65.0	69.6	<0.0001
Diabetes (%)	85.4	79.8	79.8	80.8	0.003	86.5	82.3	86.9	90.0	0.002
Total Cholesterol (mmol/l)	184.4 (40.4)	183.8 (41.0)	185.6 (42.0)	189.3 (44.0)	0.002	206.4 (44.1)	207.8 (44.5)	205.9 (44.7)	207.7 (44.0)	0.70
Triglycerides (mmol/l)	195.9 (138.7)	194.8 (124.1)	204.3 (123.3)	226.9 (148.5)	<0.0001	173.7 (94.2)	181.8 (90.8)	198.5 (119.9)	218.5 (106.3)	<0.0001
Blood glucose (mmol/l)	9.2 (3.5)	8.3 (2.9)	8.0 (2.7)	8.0 (2.7)	<0.0001	9.2 (3.8)	8.5 (3.4)	8.4 (3.1)	8.3 (3.0)	<0.0001
HgbA1c*	7.9 (1.5)	7.4 (1.3)	7.2 (1.3)	7.2 (1.3)	<0.0001	8.0 (1.6)	7.5 (1.5)	7.4 (1.4)	7.4 (1.4)	<0.0001
BMI (kg/m2)	33.2 (4.0)	33.6 (4.1)	33.7 (4.1)	34.2 (4.2)	<0.0001	34.7 (5.0)	35.2 (4.6)	36.0 (4.7)	36.3 (4.9)	<0.0001
Systolic blood pressure (mmHg)	137.5 (12.5)	137.5 (13.3)	137.5 (12.9)	137.1 (13.1)	0.82	139.5 (12.7)	139.9 (12.1)	139.2 (12.5)	138.6 (12.1)	0.10
Diast. blood pressure (mmHg)	78.3 (8.2)	78.5 (8.5)	78.3 (8.5)	78.1 (8.5)	0.73	77.2 (8.0)	77.7 (8.3)	77.7 (8.4)	76.4 (8.7)	0.0007
Diuretics users (%)	33.0	39.1	43.8	61.5	<0.0001	36.3	46.0	56.5	70.1	<0.0001
Beta-blocker users (%)	59.7	64.0	66.1	70.8	<0.0001	50.4	55.2	57.8	61.8	<0.0001
Statin users (%)	67.1	70.3	70.8	74.0	<0.0001	57.9	61.4	62.2	63.0	0.016
Aspirin users (%)	84.0	84.5	87.3	87.9	0.0005	66.1	66.3	68.6	73.6	0.0001
Sibutramine users (%)	49.9	50.0	50.0	48.7	0.57	50.9	48.5	52.1	50.6	0.68
Smokers (%)	70.7	74.9	76.2	76.4	0.0004	30.3	35.9	37.1	40.6	<0.0001
Alcohol users (%)	75.2	75.0	79.7	80.4	<0.0001	35.5	39.7	44.0	40.7	0.004
Primary outcome event (%)	12.4	10.9	12.1	14.7	0.035	7.9	6.2	7.3	11.6	0.002
All-cause mortality (%)	8.4	8.6	9.5	12.7	<0.0001	5.1	4.2	5.5	11.1	<0.0001

Continuous variables are presented as means (SD). Abbreviations: Q1 = lowest quartile: males: <317 µmol/l, females: <280 µmol/l, Q2 = second quartile: males: 317–373 µmol/l, females: 280–336 µmol/l, Q3 = third quartile: males: 373–432 µmol/l, females: 336–395 µmol/l, Q4 = highest quartile males: >432 µmol/l, females: >395 µmol/l. BMI = body mass index. Diast. = diastolic.

P-values were obtained by analysis of variance for continuous variables and by Cochran-Armitage trend test for discrete variables.

* HbA1c was only measured in diabetics.

Increasing baseline uric acid concentration was associated with increasing age, triglycerides, blood glucose and BMI in both women and men. Furthermore, increasing baseline uric acid concentration was associated with greater prevalence of pre-existing cardiovascular disease in both women and men. There was a non-linear trend towards differences in the prevalence of diabetes mellitus between SUA subgroups. Higher baseline uric acid concentrations were also associated with use of alcohol, tobacco and in particular diuretics among women and men.

### SUA and Cardiovascular Events

**Figure 1:**
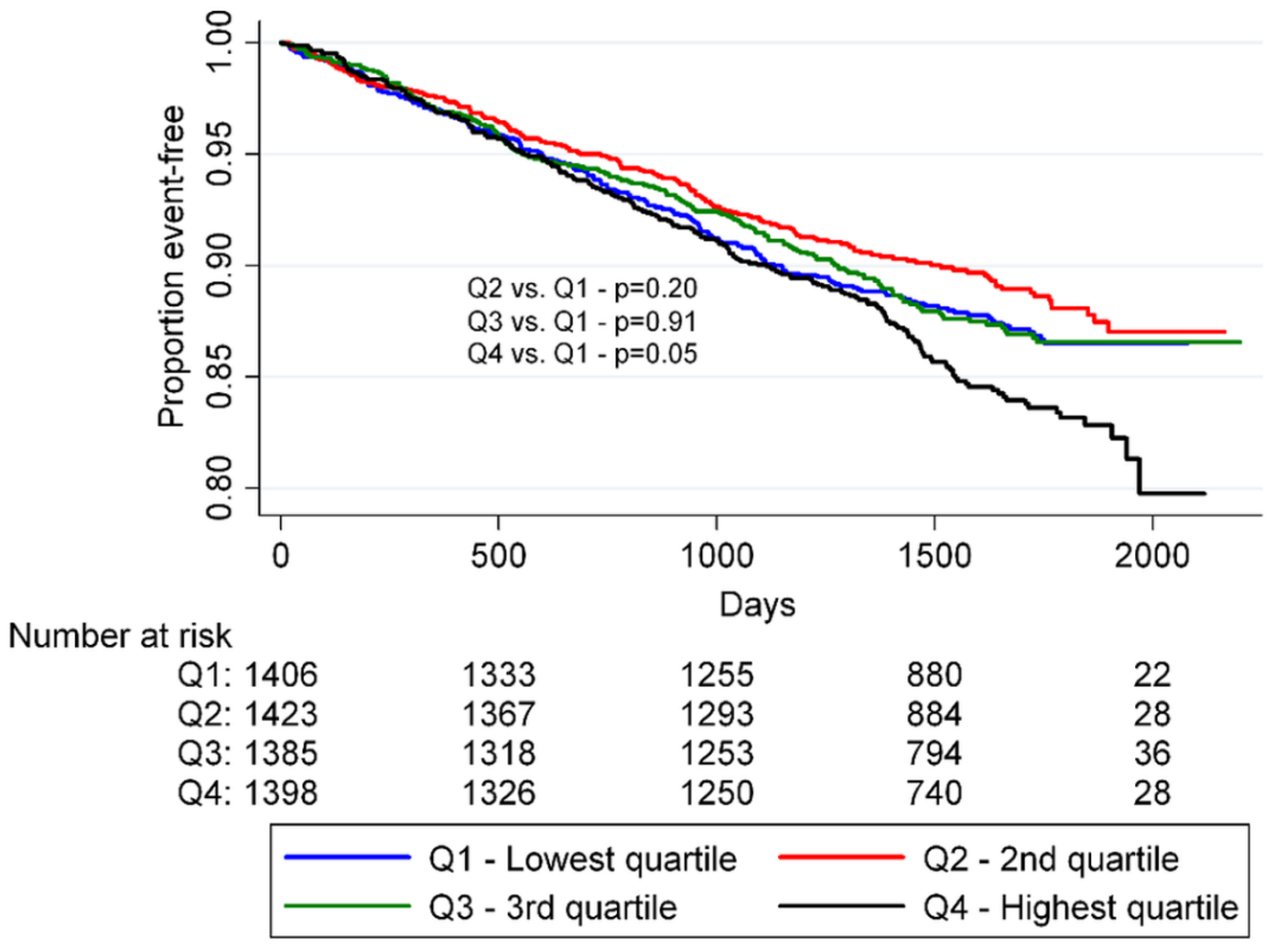
Proportion of event-free men by SUA quartiles. P-values were calculated using logrank statistics.

**Figure 2:**
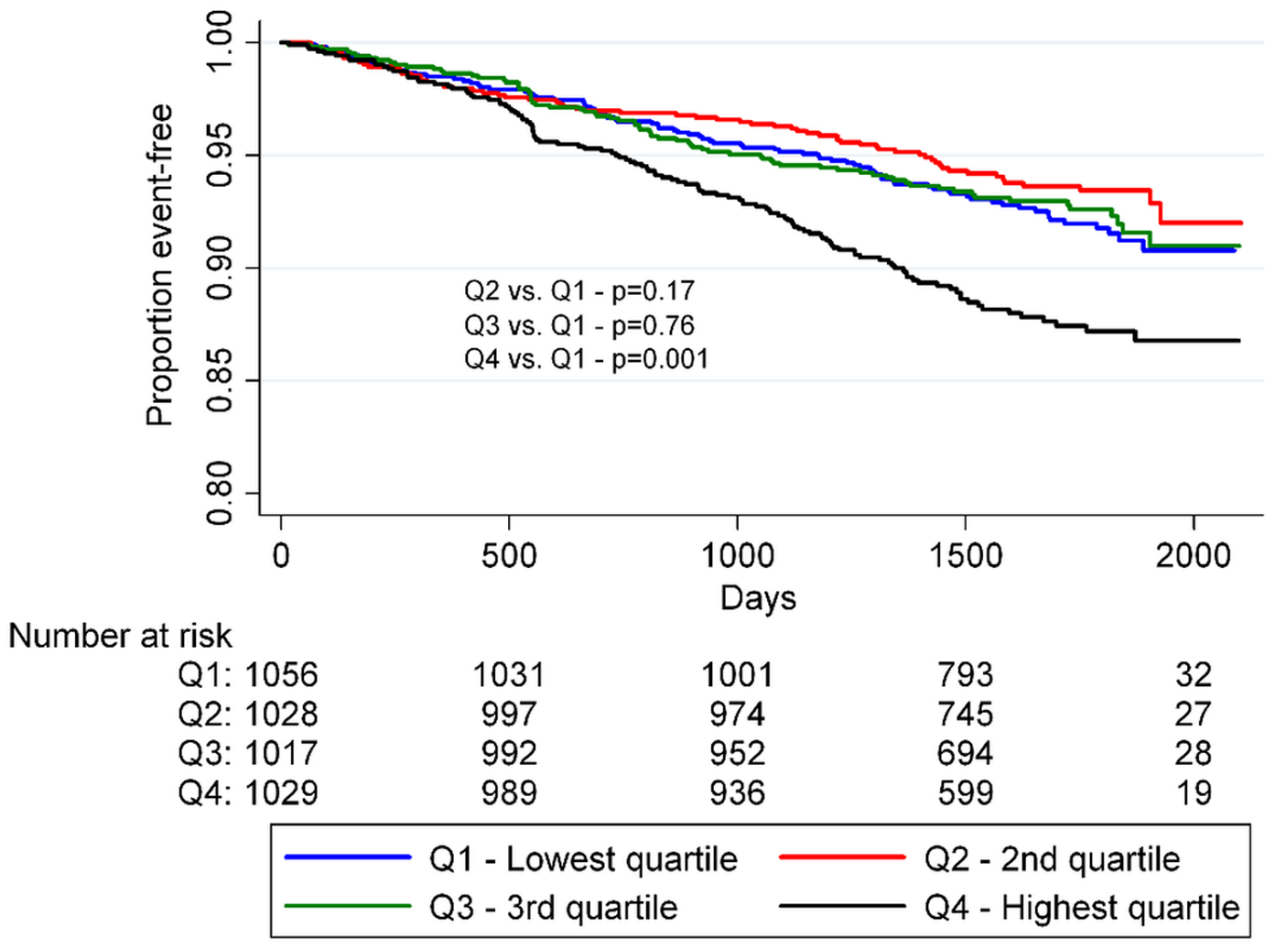
Proportion of event-free women by SUA quartiles. P-values were calculated using logrank statistics.

In the univariate Cox regression model, there tended to be a J-shaped relationship between the risk of adverse cardiovascular events and baseline SUA, with the lowest SUA quartile being the reference (Table 3). Among women the highest SUA quartile had a significantly higher risk than the reference (HR: 1.59; 95% CI: 1.20–2.10). The association between time-to-onset of primary outcome events and the SUA quartiles is further summarized in Figure 1 and 2. After more than five years of follow-up, the proportion of event-free individuals was lowest for the upper SUA quartile while it was highest for the second lowest SUA quartile (men: p = 0.05– women: p = 0.001 by the log-rank test). In a model adjusted only for age, serum creatinine and diuretic use the second lowest SUA quartile exhibited reduced risk of primary outcome events relative to the reference group for women (HR: 0.71; 95% CI: 0.51–0.98) while the increased risk in the highest SUA quartile exhibited was attenuated from (HR: 1.49; 95% CI: 1.12–1.97) to (HR: 1.03; 95% CI: 0.75–1.39).

**Table 3:** Hazard ratios and 95% confidence intervals for men and women calculated by Cox regression.

Event	SUA	Model 1	Model 2	Model 3	Model 4
**Primary outcome event, men**	Q1	1 (ref)	1 (ref)	1 (ref)	1 (ref)
	Q2	0.87 (0.70–1.08)	0.85 (0.69–1.06)	0.81 (0.65–1.01)	0.84 (0.67–1.05)
	Q3	0.99 (0.80–1.22)	0.96 (0.78–1.19)	0.88 (0.71–1.09)	0.90 (0.72–1.11)
	Q4	1.22 (1.00–1.50)	1.19 (0.97–1.46)	0.95 (0.76–1.17)	0.96 (0.77–1.20)
**Primary outcome event, women**	Q1	1 (ref)	1 (ref)	1 (ref)	1 (ref)
	Q2	0.80 (0.58–1.10)	0.78 (0.56–1.08)	**0.71 (0.51**–**0.98)**	0.73 (0.52–1.01)
	Q3	0.95 (0.70–1.30)	0.93 (0.68–1.27)	0.78 (0.56–1.07)	0.76 (0.54–1.05)
	Q4	**1.59 (1.20–2.10)**	**1.49 (1.12–1.97)**	1.03 (0.75–1.39)	0.99 (0.72–1.36)
**Cardiovascular death, men**	Q1	1 (ref)	1 (ref)	1 (ref)	1 (ref)
	Q2	1.12 (0.79–1.59)	1.09 (0.77–1.54)	1.01 (0.71–1.43)	1.01 (0.71–1.44)
	Q3	1.36 (0.97–1.90)	1.30 (0.93–1.83)	1.13 (0.81–1.59)	1.13 (0.79–1.60)
	Q4	**1.88 (1.37–2.58)**	**1.81 (1.32–2.49)**	1.28 (0.92–1.79)	1.26 (0.89–1.78)
**Cardiovascular death, women**	Q1	1 (ref)	1 (ref)	1 (ref)	1 (ref)
	Q2	**0.55 (0.31–0.99)**	**0.54 (0.30–0.97)**	**0.48 (0.26–0.86)**	**0.51 (0.28–0.92)**
	Q3	0.94 (0.57–1.56)	0.92 (0.55–1.52)	0.74 (0.44–1.24)	0.73 (0.43–1.24)
	Q4	**2.11 (1.37–3.24)**	**1.97 (1.28–3.03)**	1.28 (0.80–2.04)	1.31 (0.80–2.13)

Cut off points for quartiles: see Table 2. Ref = reference. Hazard ratios (95% confidence intervals). Primary outcome event = AMI, stroke, resuscitation after cardiac arrest or cardiovascular death.

* Hazard ratios with p-values less than 0.05 are written in bold letters.

Model 1: uric acid.

Model 2: model 1+ age.

Model 3: model 2+ baseline serum creatinine and diuretics use.

Model 4: model 3+ baseline values of cholesterol, HDL-C, LDL-C, BMI, glucose, triglyceride, history of only diabetes/both diabetes and cardiovascular disease/only cardiovascular disease, history of congestive heart failure, systolic blood pressure, diastolic blood pressure, waist-hip ratio, tobacco use, alcohol use, beta-blocker use, statin use, aspirin use and sibutramine use.

By adding each variable from the large multivariate model to the age adjusted model one at a time, we found that the increased risk originally observed in the highest quartile was no longer significant after adjusting for baseline serum creatinine concentration and use of diuretics. Therefore we created a model including SUA, age, sex, serum creatinine and diuretic use. In this model, the most hyperuricemic patients no longer had any significantly increased risk compared with the lowest quartile (Table 3), but the reduced risk among women of the second lowest quartile remained significant (HR: 0.71; 95% CI: 0.51–0.98). Further adjustment for baseline values of cholesterol, HDL-C, LDL-C, BMI, glucose, triglycerides, history of congestive heart failure, history of cardiovascular disease, history of diabetes, systolic blood pressure, diastolic blood pressure, waist-hip ratio, tobacco use, alcohol use, and randomized treatment attenuated these findings further; i.e. among women the second lowest quartile no longer exhibited reduced risk (HR: 0.73; 95% CI: 0.52–1.01) while the highest quartile exhibited similar risk (HR: 0.99; 95% CI: 0.72–1.36) to the reference group. Similar results were found for men in the final model (HR: 0.96; 95% CI: 0.77–1.20) (Table 3).

Looking at cardiovascular death as an isolated outcome, the association was stronger for the highest quartile than when considering the combined primary outcome (Table 3). It was only significant in models 1 and 2 for both women and men. However, the second quartile in female patients was associated with a lower risk in all models without obvious attenuation from model 1 through 4 (HR: 0.51; 95% CI: 0.28–0.92).

When analysing SUA as a determinant for acute myocardial infarction, there were no significant differences between SUA subgroups (HR: 0.83; 95% CI: 0.60–1.13). The same was the case for stroke (HR: 0.70; 95% CI: 0.47–1.03), resuscitated cardiac arrest (HR: 1.70; 95% CI: 0.48–6.04), and cardiovascular death (HR: 1.24; 95% CI: 0.93–1.64), even when stratifying for gender (all HRs are comparing the highest SUA quartile with the lowest SUA quartile in the fully adjusted model).

### SUA and All-cause Mortality

**Figure 3:**
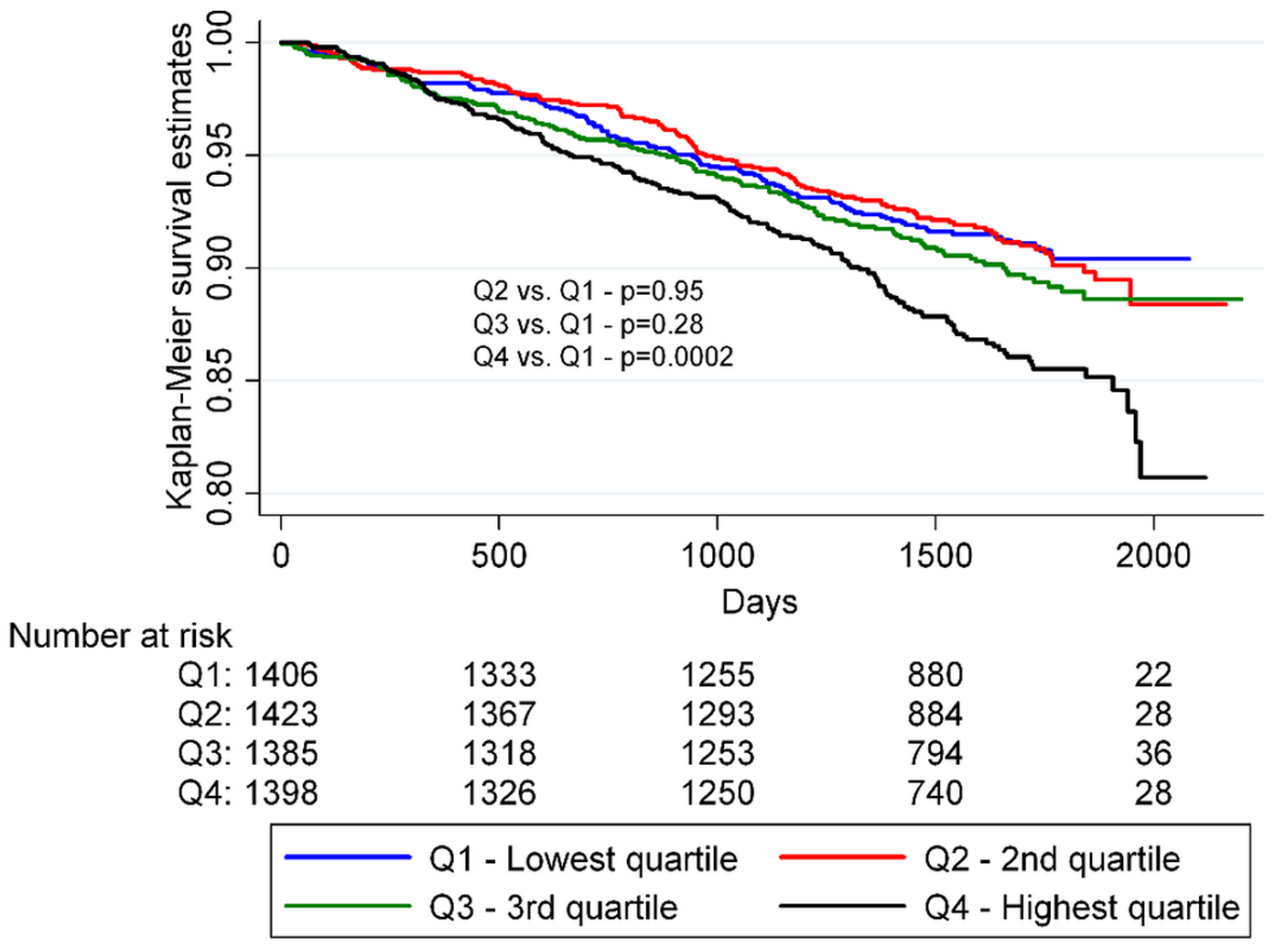
Kaplan-Meier survival analysis of men by SUA quartiles. P-values were calculated using logrank statistics.

**Figure 4:**
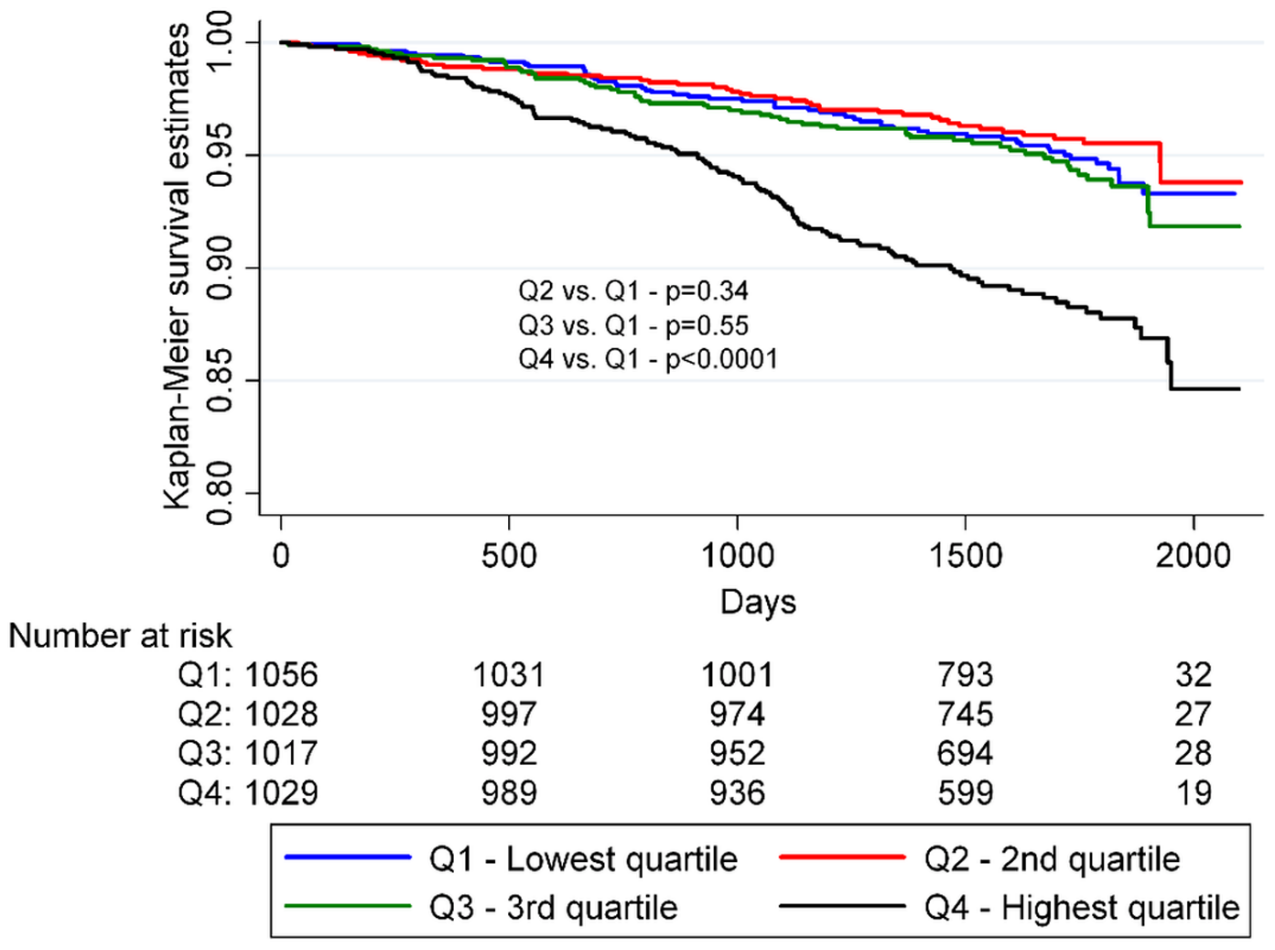
Kaplan-Meier survival analysis of women by SUA quartiles. P-values were calculated using logrank statistics.

When investigating all-cause mortality as an endpoint a significant interaction between SUA and sex was observed. Therefore we conducted a sex stratified analysis and demonstrated a stronger positive relationship between SUA and all-cause mortality in women (HR: 1.47; 95% CI: 1.02–2.12) than in men (HR: 1.10; 95% CI: 0.85–1.42) for the highest quartile compared with the lowest in the fully adjusted Cox model; p for interaction = 0.047 (Table 4). The associations between time-to-death and the SUA quartiles for men and women, respectively, are further summarized in Figure 3 and 4. We also found there was an interaction between SUA and use of sibutramine. In the placebo group SUA had no predictive value for all-cause mortality when comparing the highest and lowest quartiles in the fully adjusted Cox model (HR: 0.92; 95% CI: 0.70–1.21). In comparison a high level of SUA was associated with higher risk of all-cause mortality among patients taking sibutramine when comparing the highest and lowest quartiles in the fully adjusted Cox model (HR: 1.55; 95% CI: 1.16–2.07).

**Table 4:** Hazard ratios and 95% confidence intervals for men and women calculated by Cox regression.

Event	SUA	Model 1	Model 2	Model 3	Model 4
**All-cause mortality, men**	Q1	1 (ref)	1 (ref)	1 (ref)	1 (ref)
	Q2	1.01 (0.78**–**1.30)	0.96 (0.75**–**1.24)	0.90 (0.70**–**1.16)	0.90 (0.69**–**1.16)
	Q3	1.15 (0.90**–**1.47)	1.08 (0.84**–**1.39)	0.95 (0.74**–**1.22)	0.97 (0.75**–**1.25)
	Q4	**1.55 (1.23–1.96)** *	**1.47 (1.16–1.85)** *	1.08 (0.85**–**1.38)	1.10 (0.85**–**1.42)
**All–cause mortality, women**	Q1	1 (ref)	1 (ref)	1 (ref)	1 (ref)
	Q2	0.82 (0.55**–**1.23)	0.80 (0.54**–**1.20)	0.74 (0.49**–**1.10)	0.75 (0.50**–**1.13)
	Q3	1.11 (0.76**–**1.61)	1.08 (0.73**–**1.56)	0.93 (0.64**–**1.35)	0.88 (0.60**–**1.30)
	Q4	**2.35 (1.70–3.25)** *	**2.17 (1.57–3.00)** *	**1.62 (1.16–2.25)** *	**1.47 (1.02–2.12)** *

Cut off points for quartiles: see Table 2. Ref = reference. Hazard ratios (95% confidence intervals).

* Hazard ratios with p-values less than 0.05 are written in bold letters.

Model 1: uric acid.

Model 2: model 1+ age.

Model 3: model 2+ baseline serum creatinine and diuretics use.

Model 4: model 3+ baseline values of cholesterol, HDL-C, LDL-C, BMI, glucose, triglyceride, history of only diabetes/both diabetes and cardiovascular disease/only cardiovascular disease, history of congestive heart failure, systolic blood pressure, diastolic blood pressure, waist-hip ratio, tobacco use, alcohol use, beta-blocker use, statin use, aspirin use and sibutramine use.

### Influence of Sibutramine, BMI and Diabetes on Uric Acid Levels and Primary Outcomes

**Figure 5:**
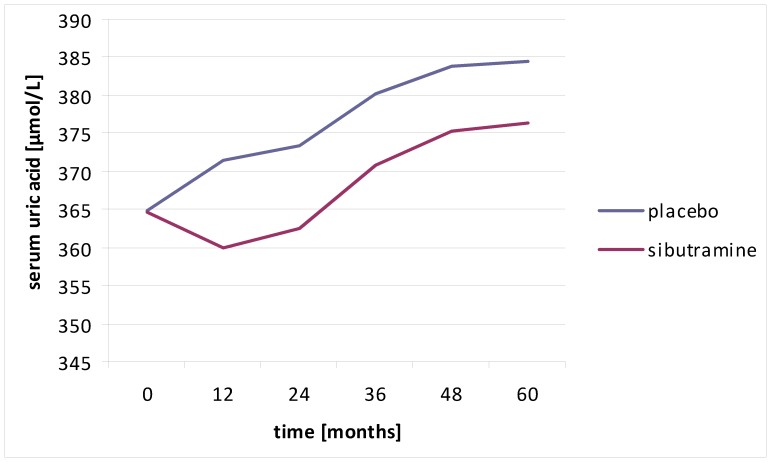
Uric acid levels over time in patients with and without use of sibutramine.

Use of sibutramine was found to be associated with a mean fall in uric acid levels as presented in Figure 5. Because of this potential change of uric acid levels with time we also performed a time dependent proportional hazard analysis where uric acid was updated at each yearly visit. This analysis provided similar results to those described above. Hazard ratios and 95% CIs of the 3 upper quartiles relative to the lowest quartile were: 0.95 (0.75–1.20), 0.96 (0.76–1.22), and 1.12 (0.90–1.40). These should be compared to the results of model 4 in Table 3. We investigated for interactions between use of sibutramine and sex-stratified uric acid groups and found that they were insignificant (p = 0.55 and 0.81 for all-cause mortality and for the primary endpoint, respectively).

Exploring the relationship between changes in BMI and uric acid (changes in relation to baseline values), a differential effect of a loss in BMI was found for the first and second year between patients with and without diabetes, where patients without diabetes were found to have greater falls in uric acid for each 1 kg/m^2^ fall in BMI, compared to patients with diabetes (3.6± standard error [SE] 1.2 µmol/l vs. 2.9±0.5 µmol/l, p = 0.03 for year 1; and 6.4±1.2 µmol/l vs. 3.4±0.5 µmol/l, p = 0.006 for year 2), but not for year 3 and 4 (4.6±1.3 µmol/l vs. 4.2±0.6 µmol/l, p = 0.6 for year 3; and 3.9±1.3 µmol/l vs. 4.6±0.7 µmol/l, p = 0.6 for year 4).

## Discussion

In this population of nearly 10,000 obese/overweight subjects there was an apparent J-shaped relationship between baseline SUA and risk of cardiovascular adverse events, but when adjusted for other cardiovascular risk factors the increased risk for the highest uric acid quartile was attenuated. The lower risk for the second lowest quartile remained significant after adjustment for various confounders among women. This could be because uric acid is also partly a marker of metabolic state. In this context it is interesting to notice that an increasing level of SUA is correlated with many other cardiovascular risk factors such as dyslipidemia and previous myocardial infarction.

Our previous study investigated the association between changes in fasting blood glucose and changes in SUA in the four week lead-in period of the SCOUT trial [17]. The change in SUA was found to differ according to the presence of diabetes. In patients with diabetes, a decrease of 1 mmol/L in fasting serum glucose was associated with only a 1.7±0.3 mmol/L fall in SUA, whereas in the non-diabetes group each 1 mmol/L decrease in fasting blood glucose was associated with a 3.7±1.5 mmol/L larger fall in SUA. Sibutramine itself also seemed to promote a greater fall in uric acid levels than that predicted from weight loss alone putatively by inhibiting the SLC2A9 uric acid kidney tubular transporter where uric acid reabsorption also interacts with glucose reabsorption [17], [18]. Thus poorly controlled patients with diabetes, with higher blood and urinary glucose levels have a greater fall in blood uric acid levels because of the exchange of intracellular uric acid for tubular glucose in the apical glucose transporter part of the SLC2A9 transporter. This could partly account for our findings of paradoxically increased risk of adverse outcomes associated with very low uric acid concentrations (if very low uric acid concentrations were a marker of poor diabetes control and severe glucosuria). Indeed we could identify an initial (for the first two years) greater fall in uric acid for each 1 kg/m^2^ loss in BMI for patients without diabetes, compared with patients with diabetes.

Because of this effect modifying feature of diabetes we tested for interactions between both diabetes status and fasting blood glucose and SUA on the risk of primary outcome. We also would have liked to test the interaction with HbA1c (generally seen as a better measure of diabetes control), but this was only measured in patients with diabetes. However, these interaction tests were all insignificant, and therefore, we did not stratify further. In the diabetes group, there was no interaction between HbA1c and SUA.

We found a gender difference in the impact of SUA on all-cause mortality. For both genders there was a J-shaped association between SUA and all-cause mortality, but the increased risk of the highest uric acid quartile remained significant in the large multivariate Cox model in women only. Previous studies have indicated that loop diuretics may influence uric acid levels. Hence, loop diuretics rather than uric acid levels may carry an increased risk of adverse outcomes [19]. This was also observed in our analyses, where adding creatinine and diuretics to our Cox models attenuated the estimates.

Interestingly, previous data have suggested that uric acid metabolism is more resistant to the influence of fructose intakes in women than in men, which could suggest that comparable elevations in uric acid levels are likely to mirror a rather more severe metabolic disturbance in women than in men.

Similar results have been obtained by Ong et al. [8] who investigated SUA as an independent risk factor for cardiovascular disease and death and all-cause mortality in a cohort of 1294 patients with type 2 diabetes in the Fremantle Diabetes Study. They did not find that SUA could predict these outcomes after adjustment for relevant confounders. This was regardless of whether SUA was considered a continuous or discrete (quartiles) covariate in the Cox proportional hazard models. In our cohort more than 80% of subjects had diabetes and the age distribution was similar to the Freemantle Diabetes Study making the two studies comparable, though our cohort included a larger proportion of men and was much larger in size.

Our finding of a lack of an independent association between baseline SUA and primary outcome events is in contrast to several other studies on uric acid and cardiovascular risk. The American study NHANES I was a population-based study, including nearly 6000 subjects and with a very long follow-up period of more than 16 years. They found that increased levels of serum uric acid had a positive relationship to cardiovascular mortality in men and woman and in both black and white ethnic groups [2]. It should, however, be emphasized that, although our study population included nearly 10,000 patients at high risk of cardiovascular events, the myocardial infarction endpoint on its own may have been underpowered to confirm a true relationship.

In the very large Swedish AMORIS study (n = 417,734) which was a register based prospective study on AMI, congestive heart failure (CHF) and stroke in a population without prior cardiovascular disease, a gradual increase in risk of AMI, CHF, and stroke was found with increasing SUA levels, suggesting that SUA is an important complementary indicator of cardiovascular risk in the general population [3]. It is also intriguing that two patient cohorts [11], [12] with different degrees of uric acid lowering with allopurinol also showed significant reductions in cardiovascular events with more intensive treatment. This implies that if there is an independent effect of uric acid evident in large population studies of reasonably healthy people then the impact of uric acid per se in patients at high cardiovascular risk is overwhelmed by the importance of the other metabolic risk factors with which uric acid increases are associated. However, a more likely explanation is that allopurinol, a xanthine oxidase has other ‘off target’ effects as shown by its antioxidant properties and its role in increasing endothelium-dependent vasodilation and prolonging the time to angina on exercise [20], [21].

Strengths of our study included the large number of participants, and a well-defined cohort of overweight patients. We also had information on many possible confounders, such as smoking, BMI, diuretics use, and serum creatinine. However, the present analysis represents a retrospective evaluation of a prospective clinical trial and, as a consequence, is subject to the limitations of such an approach.

A clear limitation is the lack of information on glomerular filtration rate and serum insulin, since these variables could be affected in patients with cardiovascular disease, and they can also affect the level of SUA [22]. We also lacked information on use of drugs to treat hyperuricemia, such as allopurinol. Further we did not have information on fructose intake which recently has been suggested to have a causal influence on uric acid levels (i.e. a high fructose intake may increase uric acid levels) [23].

We have shown that sibutramine lowers SUA levels [17], but despite controlling for sibutramine use we cannot fully rule out a treatment effect on the lack of association between SUA levels and CV mortality observed in this study.

In conclusion, our study did not show serum uric acid to be an independent risk factor of cardiovascular events and death in a population of high cardiovascular risk overweight/obese subjects. However, our results suggested that SUA was an independent predictor of all-cause mortality in women.
